# Biceps-based 3-layer reconstruction of the irreparable rotator cuff: a technical note on biceps tendon as a common local graft for in-situ superior capsular reconstruction, partial cuff repair, and middle trapezius tendon transfer

**DOI:** 10.1186/s13018-023-03978-0

**Published:** 2023-07-15

**Authors:** Amr Abdel-Mordy Kandeel

**Affiliations:** Department of Orthopedics and Traumatology, Faculty of Medicine, Menoufia University, Gamal Abdel-Nasser Street, Shebien El-kom, Menoufia Governorate Egypt

**Keywords:** Irreparable rotator cuff tears, Long head of biceps tendon, Middle trapezius tendon transfer, Partial rotator cuff repair, Rotator cuff re-tear, Superior capsular reconstruction, Tendon transfer for irreparable cuff tears

## Abstract

**Background:**

For irreparable rotator cuff tears, 3-layer tendon reconstruction (in which in-situ superior capsular reconstruction-reinforced partial rotator cuff repair was augmented with hamstring-sheet-lengthened middle trapezius tendon transfer) was recently reported to achieve satisfactory postoperative outcomes. To avoid hamstring graft-related drawbacks, the current note describes a technical modification of that reconstruct; wherein long head of biceps tendon is used as a cornerstone structure for simultaneously reconstructing the superior capsule; lengthening the transferred middle trapezius tendon; and augmenting the partially-repaired rotator cuff.

**Methods:**

Via sub-pectoral approach, long head of biceps tendon is distally-tenotomized. Through McKenzie approach, proximal stump of the tenotomized long head of biceps is retrieved to the sub-acromial space where double-row biceps tenodesis (into a trough at the greater tuberosity) is performed for reconstructing the superior capsule. Next, postero-superior rotator cuff is partially repaired, and side-to-side sutured to the reconstructed capsule. Through a 7–8-cm skin incision over the medial scapular spine, middle trapezius tendon is released. Portion of long head of biceps tendon distal to the tenodesis site is retrieved via a sub-trapezius/sub-acromial corridor to the scapular wound where it is re-attached to the released middle trapezius tendon.

**Results:**

Use of long head of biceps tendon as a common local graft (for simultaneously reconstructing the superior capsule, lengthening the transferred middle trapezius tendon, and augmenting the partially-repaired rotator cuff) is technically feasible provided that the harvested tendon stump is at least 10 cm in length.

**Conclusion:**

While avoiding hamstring graft-related complications, the currently-reported biceps-based 3-layer rotator cuff tendon reconstruction might offer the advantages of reproducibility, safety, simplicity and quickness; however, it should be validated via further studies.

*Trial registration* The present study was approved by the Institutional Committee of Scientific Research and Ethics (3-2023Orth10-1).

**Supplementary Information:**

The online version contains supplementary material available at 10.1186/s13018-023-03978-0.

## Introduction

Currently, in-situ (i.e., long head of biceps [LHB] tendon-based) superior capsular reconstruction (SCR) and tendon transfer are among the most commonly exercised modalities for management of irreparable postero-superior rotator cuff (RC) tears [1–10].

Biomechanically, in-situ SCR has been shown to be effective in restraining superior migration of the humeral head via check-rein and sub-acromial spacer mechanisms; however, the static nature of in-situ SCR remains a major demerit [3, 11].

On the other hand, due to its dynamic nature, tendon transfer (e.g., latissimus dorsi) represents a more attractive management option. However, long-term sustainability of humeral head re-centralization over the glenoid following that tendon transfer is still questionable [6, 9].

To overcome the latter shortcoming of tendon transfer; Kandeel has lately introduced the transfer of medial (lower) portion of the middle trapezius tendon (MTT) for dynamic functional reproduction of the supraspinatus (SSP), which in turn is to result in effective re-centralization of the humeral head over the glenoid [12].

The latter rationale of MTT transfer was investigated in a recently-published cohort study of irreparable postero-superior RC tears, in which Kandeel concluded that a three-layer tendon reconstruct (i.e., augmentation of partially-repaired cuff, on its articular side, with in-situ SCR; and on its bursal side, with hamstring-tendon-lengthened MTT transfer) has shown more superior postoperative functional outcomes compared with a two-layer tendon reconstruct (i.e., in-situ SCR-reinforced partial RC repair) [13].

However, a major technical default of MTT transfer is the need for an intervening sheet of hamstring tendons auto-graft to lengthen the transferred tendon to the native RC footprint. Hamstring graft-related drawbacks such as prolonged operative time, troublesome setup/patient positioning, higher risk of infection and donor site morbidity might hinder popularization of that currently-evolving MTT transfer [13].

**Fig. 1:**
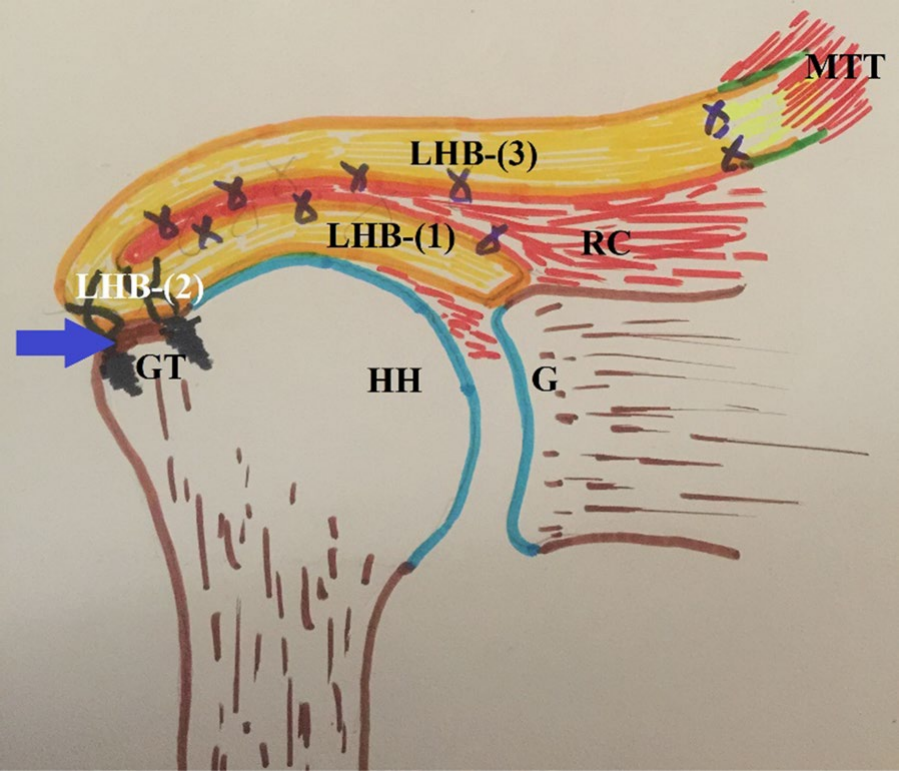
Demonstrates the technical principle of the currently-reported technique of biceps-based three-layer rotator cuff reconstruction in a right shoulder; in which long head of biceps tendon is simultaneously used as a local graft for the in-situ superior capsular reconstruction; an interposition sheet (as an alternate to the hamstring tendon autograft) to lengthen the transferred middle trapezius tendon to the cuff footprint; and an anchorage structure for the partially-repaired rotator cuff. According to the current technical note, proximal stump of long head of biceps tendon (following distal/sub-pectoral tenotomy) can be divided into three segments: LHB-(1); the proximal segment (2.5 cm) utilized for in-situ superior capsular reconstruction, and anchorage of the partially-repaired postero-superior rotator cuff; LHB-(2); the middle segment (1.5–2 cm) utilized for double-row biceps tenodesis into a (blue arrow-marked) trough at the grater tuberosity; and LHB-(3); the distal segment (7–7.5) utilized as a lengthening graft for the transferred middle trapezius tendon; G, glenoid; GT, greater tuberosity; HH, humeral head; LHB, long head of biceps; MTTT; middle trapezius tendon; RC, rotator cuff

For technical simplification and effective reproducibility of that three-layer tendon reconstruct, the current note describes a biceps-based three-layer RC reconstruction in which LHB tendon is simultaneously used as a local graft for in-situ SCR; an interposition sheet (as an alternate to the hamstring tendons autograft) to lengthen the transferred MTT to the cuff footprint; and an additional anchorage structure for partial RC repair. Figure 1 demonstrates the technical principle of the currently-reported technique of biceps-based three-layer RC reconstruction.

## Operative technique

The currently reported note was approved by the Institutional Review Board as a reconstructive technique for primary and revision management of irreparable postero-superior RC tears in relatively-young active population with high functional demands.

### Setup

**Fig. 2:**
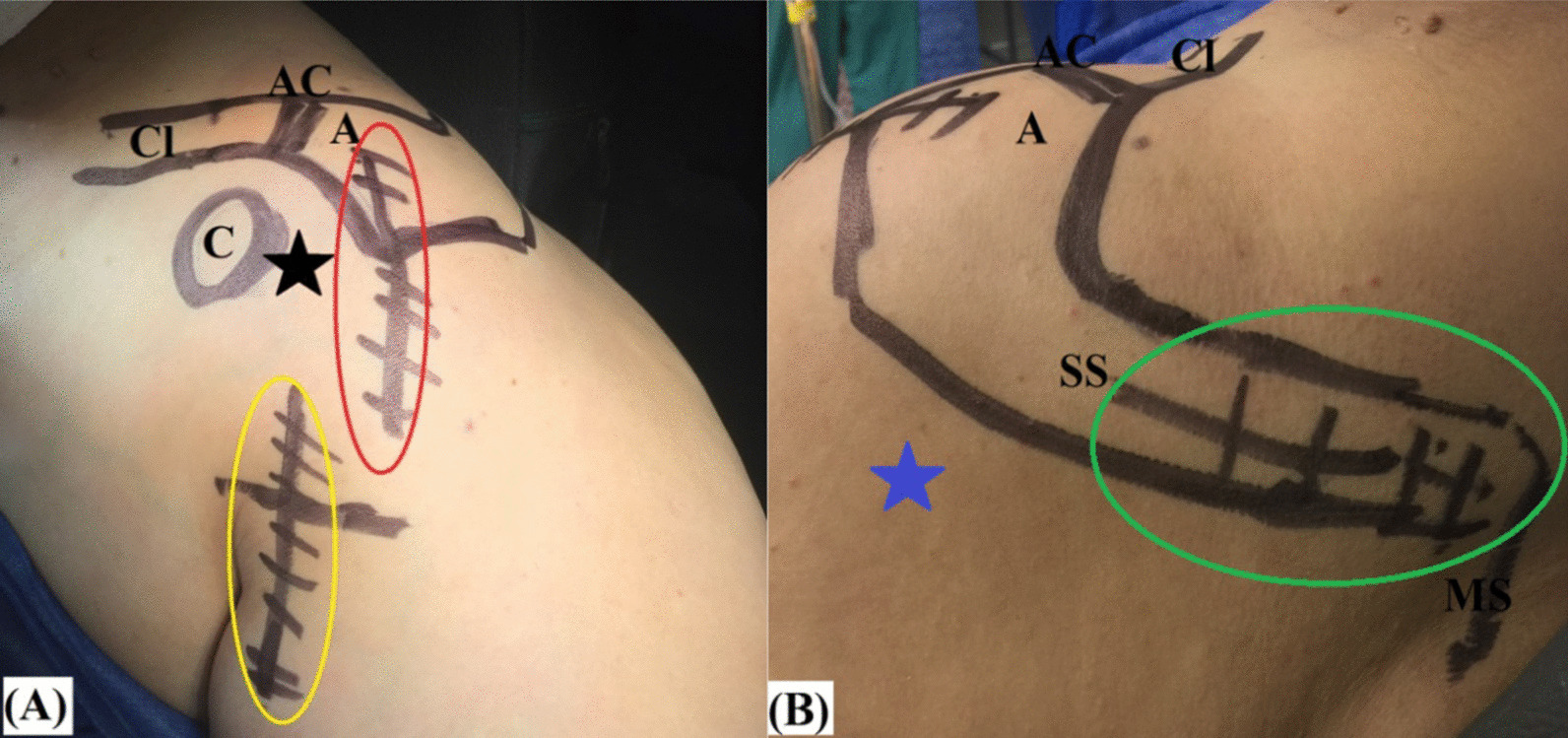
**A**, **B** Demonstrates the pen-marked anatomic landmarks, arthroscopic portals and surgical approaches of the currently reported technique of biceps-based 3-layer rotator cuff reconstruction in a left shoulder while seating the patient in a beach-chair position. **A** Anterior aspect of the shoulder; **B** posterior aspect of the shoulder. A, the acromion; AC, the acromio-clavicular joint; C, the coracoid process; Cl, the clavicle (lateral end); MS, the medial scapular border, SS, the scapular spine; the black star, the anterior mid-glenoid arthroscopic portal; the blue star, the posterior arthroscopic portal; the red oval marks the McKenzie approach; the yellow oval marks the sub-pectoral approach; the green oval marks the scapular approach

Following general anesthesia, antibiotic prophylaxis (intra-venous administration of 1gm of Meropenem), beach-chair positioning, and pen-marking of the related anatomic landmarks, passive range of motion (ROM) of the operated shoulder is assessed for exclusion of associated frozen shoulder. Figure 2A, B demonstrates the pen-marked anatomic landmarks, arthroscopic portals and surgical approaches of the currently reported technique of biceps-based 3-layer RC reconstruction.

### Diagnostic arthroscopic gleno-humeral (GH) examination

Via standard posterior and anterior mid-glenoid portals, arthroscopic GH examination is performed to confirm the preoperative diagnosis of RC irreparability (i.e., massive retracted tear with poor soft tissue quality of the tendon stump); to ascertain intact/reparable subscapularis (SSC) tendon; assess integrity of the labral attachment/intra-articular portion of LHB tendon; and preclude concomitant intra-articular GH pathology (e.g., arthritic changes).

### The McKenzie approach

Through a 4–5-cm-long skin incision (starting from the acromioclavicular [AC] joint, extending obliquely toward the antero-lateral corner of the acromion, and ending at a point located at a distance of 3–4 cm distal to this corner), the subcutaneous tissue is peeled off with a dry gauze sponge exposing the deltoid raphe. The latter is longitudinally-divided allowing access into the sub-acromial space between the anterior one and the posterior two thirds of the deltoid muscle.

To facilitate future biceps-based three-layer RC reconstruction, the sub-acromial space is decompressed (i.e., bursectomy, release of the coracoacromial ligament, anterior acromioplasty and debridement of the arthritic AC joint).

### The sub-pectoral approach

Through a 4–5-cm vertical skin incision centered at intersection point of the inferior border of pectoralis major (PM) and the medial border of biceps brachii, subcutaneous tissue and fascia are incised in order to expose the inferior border of PM which is then gently-retracted superiorly using a retractor. With blunt dissection (e.g., using an artery clamp), sub-pectoral space is explored till identification of LHB tendon which can be rolled with the surgeon’s fingertip against the humerus.

**Fig. 3:**
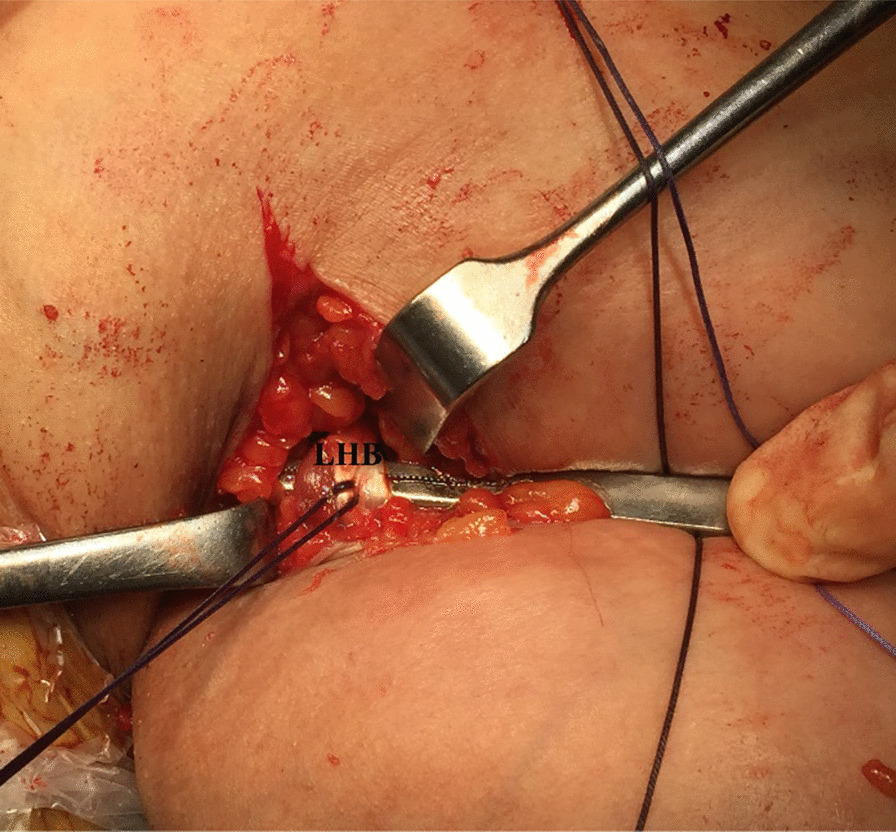
Demonstrates sub-pectoral identification and suture tagging of the long head of biceps tendon in a left shoulder while seating the patient in a beach-chair position. Following sub-pectoral identification and #2 absorbable suture tagging, the long head of biceps tendon is cleaned off from the surrounding soft tissues along down the tendon as distal as possible to maximize the length of the harvested tendon; the latter step can be further facilitated with placing the ipsilateral elbow in > 90° flexed position; and with upward traction over the tagging sutures. Then, the tendon is tenotomized distal to the tagging sutures. LHB, long head of biceps

Following identification, the LHB tendon is lifted up over a curved artery clamp; tagged with #2 absorbable sutures (Vicryl, Ethicon, Cincinnati, OH, USA); and cleaned off from the surrounding soft tissues along down the tendon as distally as possible to maximize the length of the harvested tendon; the latter step can be further facilitated with placing the ipsilateral elbow in > 90° flexed position; and with upward traction over the tagging sutures. Then, the LHB tendon is tenotomized distal to the tagging sutures. Figure 3 demonstrates sub-pectoral identification and suture tagging of the LHB tendon prior to its distal (sub-pectoral) tenotomy. The LHB tendon distal to the tenotomy site is re-attached to the nearby PM using #2 absorbable sutures.

**Fig. 4:**
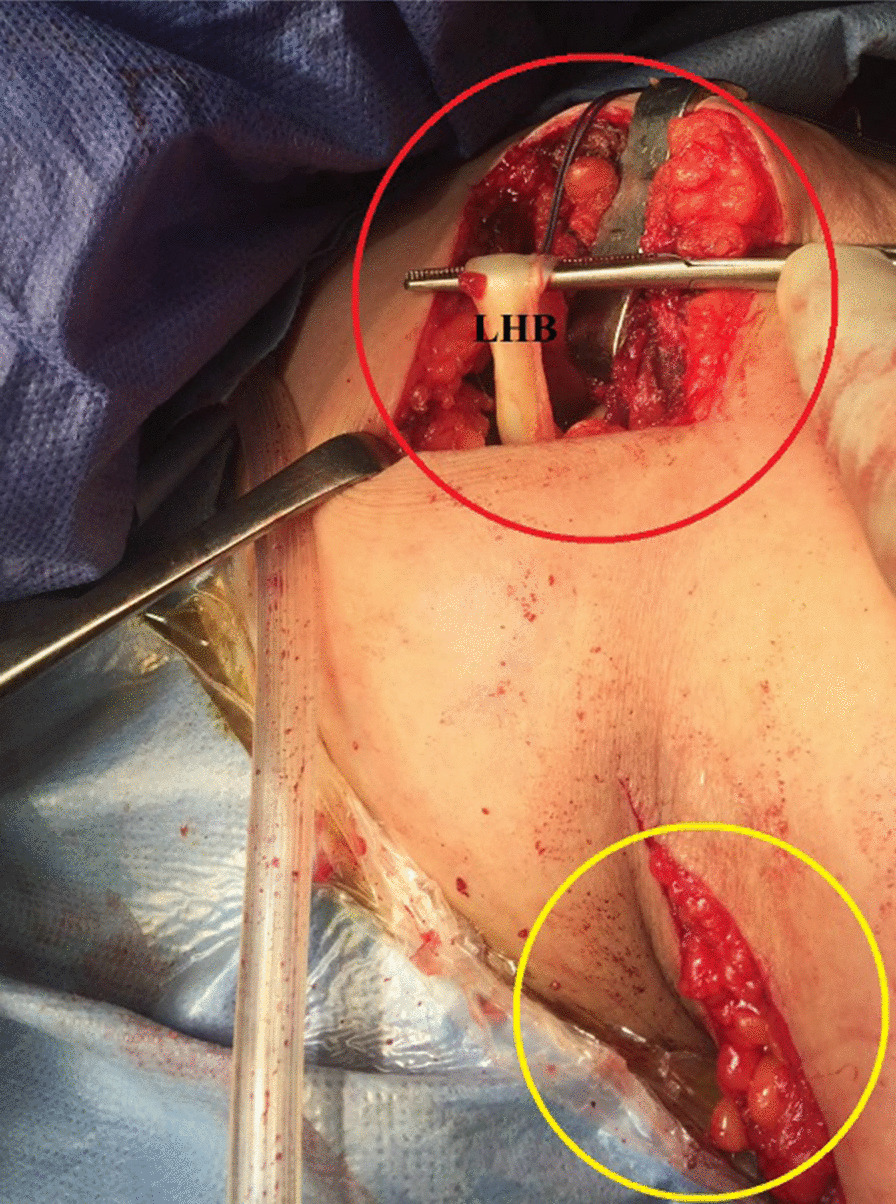
Demonstrates sub-acromial retrieval of the proximal stump of the distally-tenotomized long head of biceps tendon in a left shoulder while seating the patient in a beach-chair position. Following its distal (sub-pectoral) tenotomy, the proximal stump of the distally-tenotomized long head of biceps tendon is then retrieved from the (yellow circle-marked) sub-pectoral region to the (red circle-marked) sub-acromial space using an artery clamp. LHB, long head of biceps

Using a long straight artery clamp, the proximal stump of the distally-tenotomized LHB tendon is then retrieved from the sub-pectoral region to the sub-acromial space. Figure 4 demonstrates sub-acromial retrieval of the proximal stump of the distally-tenotomized LHB tendon.

### The 1st layer of the reconstruct: in-situ (LHB tendon-based) SCR

**Fig. 5:**
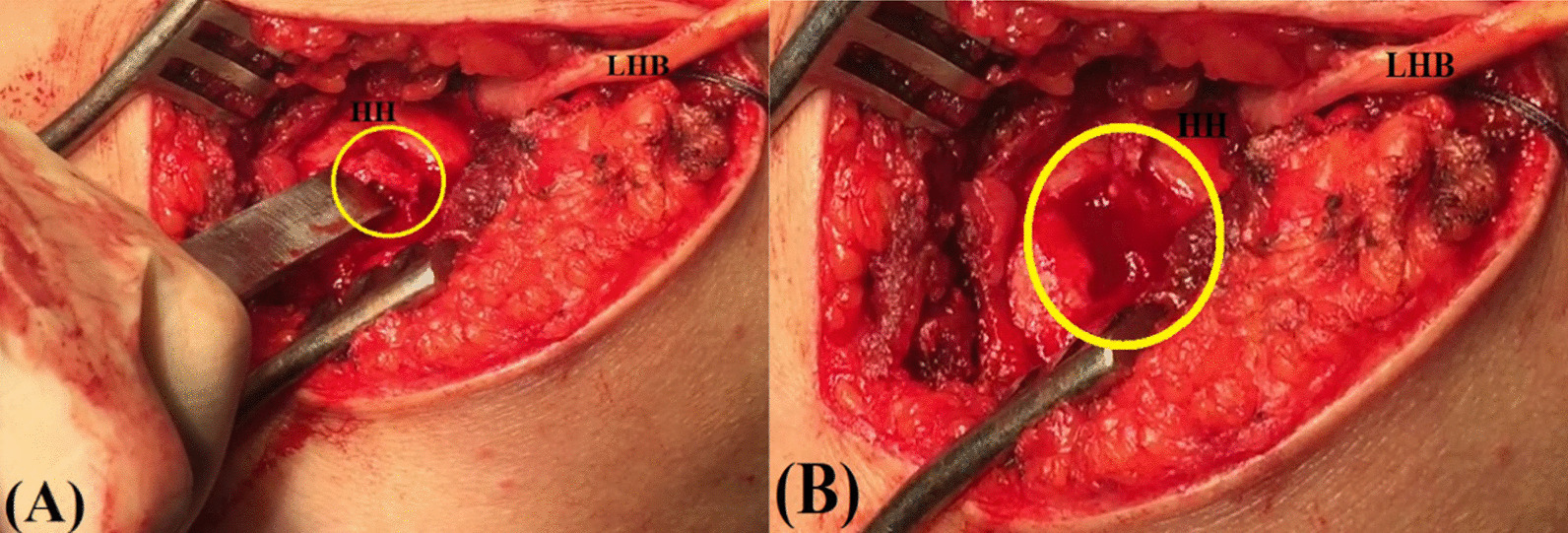
Demonstrates a trough created over the greater tuberosity in a left shoulder while seating the patient in a beach-chair position. **A** An osteotome and a mallet are used to create a (yellow circle-marked) trough at the mid-portion of the greater tuberosity starting just lateral to the articular margin of the humeral head; **B** dimensions of the (yellow circle-marked) trough should be at least 1.5–2 cm in length, 1 cm in width and 0.5 cm in depth in order to adequately accommodate the transposed LHB tendon. HH, humeral head; LHB, long head of biceps

Afterwards, starting from just lateral to the articular margin of the humeral head, a motorized burr (or alternately, an osteotome and a mallet) is used to create a 1.5-2 cm long vertical trough over the mid-portion of the greater tuberosity. Figure 5A, B demonstrates a trough created over the greater tuberosity.

Then, a 5-mm double-loaded titanium suture anchor (Corkscrew, Arthrex, Naples, FL, USA) is inserted into proximal half of the trough. A direct suture passer (Bird-peak, Arthrex, Naples, FL, USA) is used to pass a limb of each suture through the LHB tendon. While placing the shoulder in 30°-abduction/0°-rotation, the sutures are tied using Fisherman sliding knot secured with further 4 alternating half hitches. Free suture limbs are left uncut to be used in the following partial RC repair.

**Fig. 6:**
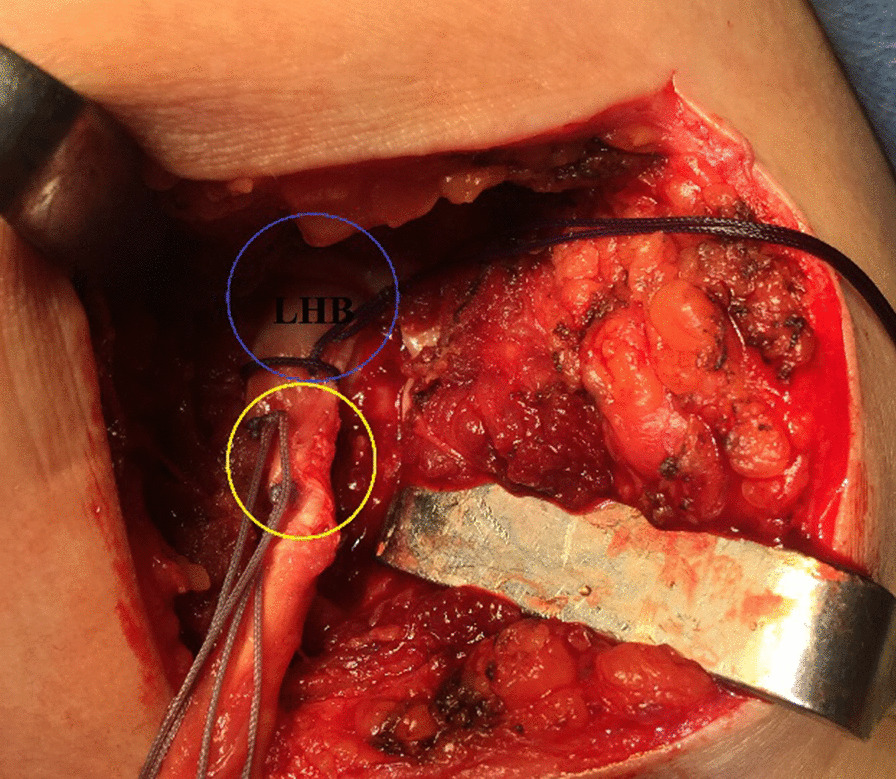
Demonstrates the long head of biceps tendon-based superior capsular reconstruction in a left shoulder while seating the patient in a beach-chair position. Using (yellow circle-marked) 2 suture anchors inserted sequentially into the proximal and distal portions of the trough, the long head of biceps tendon is transposed and affixed into the trough for structural/functional reconstitution of the (blue circle-marked) superior gleno-humeral capsule. LHB, long head of biceps

Using another 5-mm double-loaded suture anchor inserted into distal half of the trough, the previous steps are repeated in order to complete a double-row biceps tenodesis for structural and functional reconstitution of the superior GH capsule taking advantage of the proximal segment (2.5 cm) of the LHB tendon while leaving its portion distal to the tenodesis site (7–7.5 cm) as a free segment for future use in LHB tendon-based MTT transfer. Figure 6 demonstrates the LHB tendon-based SCR.

### The 2nd layer of the reconstruct: LHB tendon-based partial RC repair

Thereafter, RC footprint is debrided and minimally decorticated. A #2 absorbable traction suture is passed through the retracted postero-superior cuff to ease its mobilization and release (with blunt dissection, i.e., the surgeon index finger/a dry gauze sponge) from the surrounding adhesions. Taking advantage of uncut free suture limbs of the anchors used for biceps tenodesis, partial postero-superior RC repair is then accomplished.

**Fig. 7:**
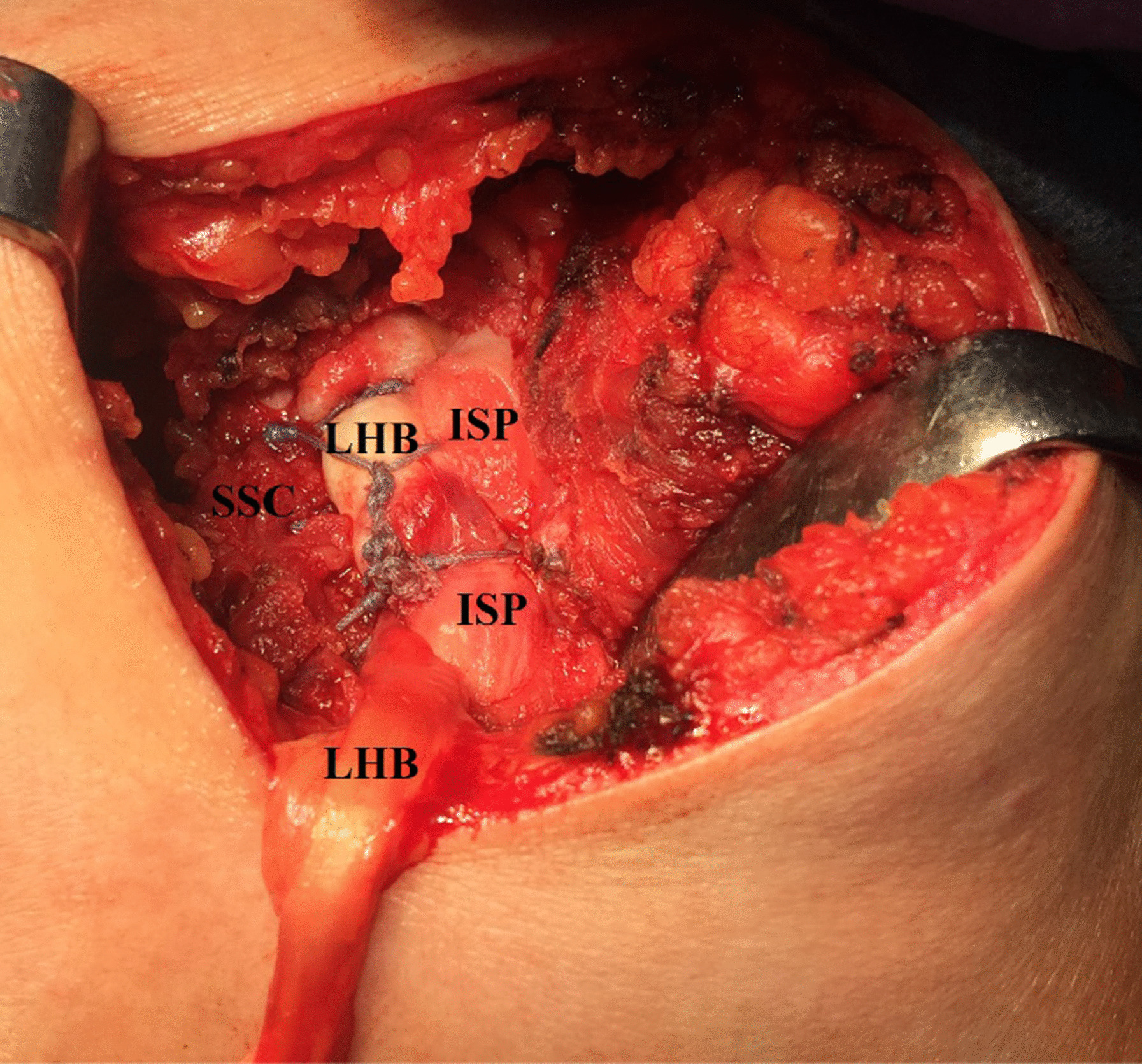
Demonstrates long head of biceps tendon-based partial rotator cuff repair in a left shoulder while seating the patient in a beach-chair position. Taking advantage of the suture limbs of the suture anchors used for in-situ superior capsular reconstruction, postero-superior cuff is partially repaired; for re-enforcement of this repair, 3–4 side-to-side simple stitches are performed annexing the long head of biceps tendon and the partially-repaired cuff together. When evident, concurrent subscapularis tear is repaired using a 3rd suture anchor; the repaired subscapularis is further side-to-side sutured to the long head of biceps tendon. ISP, infraspinatus tendon; LHB, long head of biceps; SSC, subscapularis tendon

For re-enforcement of RC repair, 3–4 side-to-side simple stitches (using absorbable sutures) are performed annexing the LHB tendon and the partially-repaired cuff together. Figure 7 demonstrates the LHB tendon-based partial RC repair.

When evident, concurrent SSC tear is repaired via simple stitches using a 3rd 5-mm suture anchor. The repaired SSC is then side-to-side sutured to the LHB tendon.

### The 3rd layer of the reconstruct: LHB tendon-based MTT transfer

**Fig. 8:**
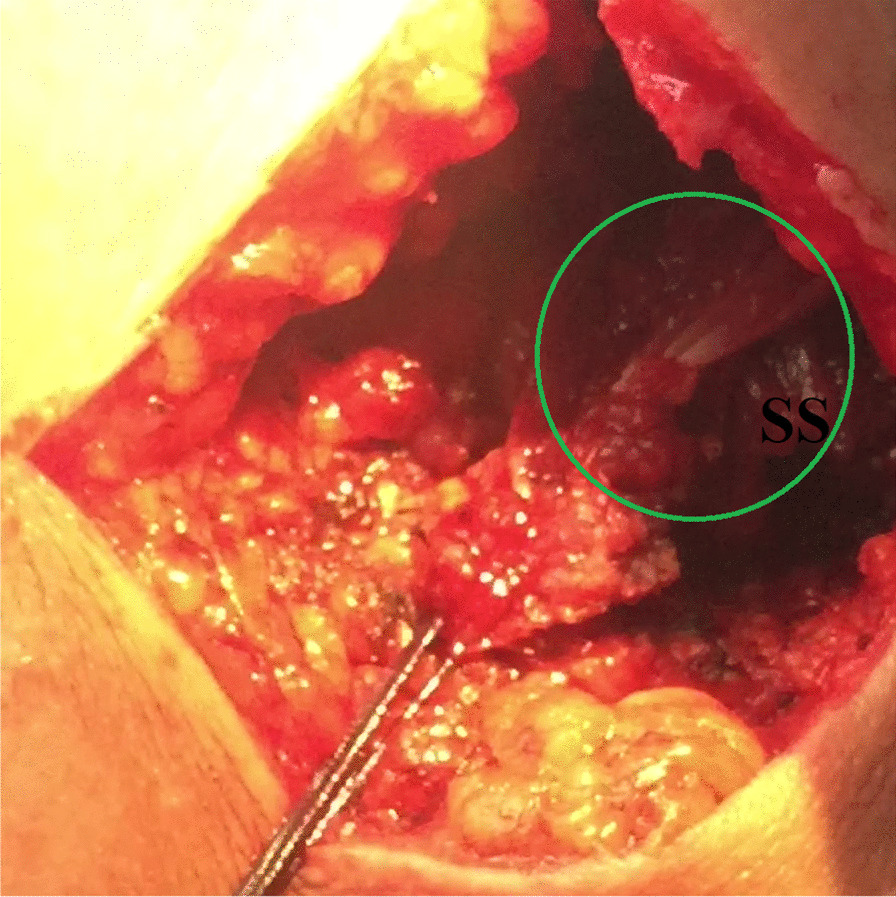
Demonstrates the released medial portion of the middle trapezius tendon in a left shoulder while seating the patient in a beach-chair position. Through a 7–8-cm-long skin incision (along the medial portion of the scapular spine), the (green circle-marked) insertion tendon of the medial portion of the middle trapezius muscle is identified, tagged with #2 absorbable traction sutures, released from the scapular spine (using a diathermy probe), and freed from the underlying atrophied supraspinatus muscle (in order to maximize excursion of the released tendon). SS, scapular spine

**Fig. 9:**
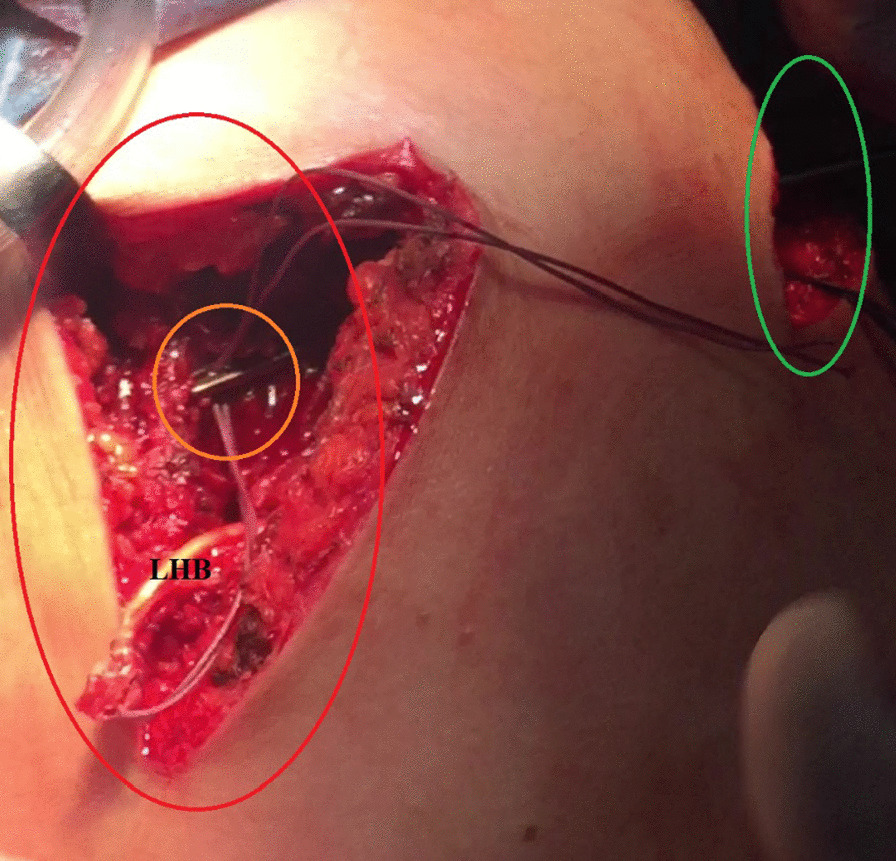
Demonstrates sub-acromial/sub-trapezius retrieval of the free segment of the long head of biceps tendon from the sub-acromial space to the scapular wound in a left shoulder while seating the patient in a beach-chair position. Following release of insertion tendon of the medial portion of the middle trapezius muscle, a long straight artery clamp (marked in an orange circle) is then passed through the (green oval-marked) scapular approach running above the partially repaired rotator cuff to appear at the (red oval-marked) sub-acromial space to establish a sub-acromial/sub-trapezius corridor for retrieving the free segment of the long head of biceps tendon from the sub-acromial space to the scapular wound. LHB, long head of biceps

**Fig. 10:**
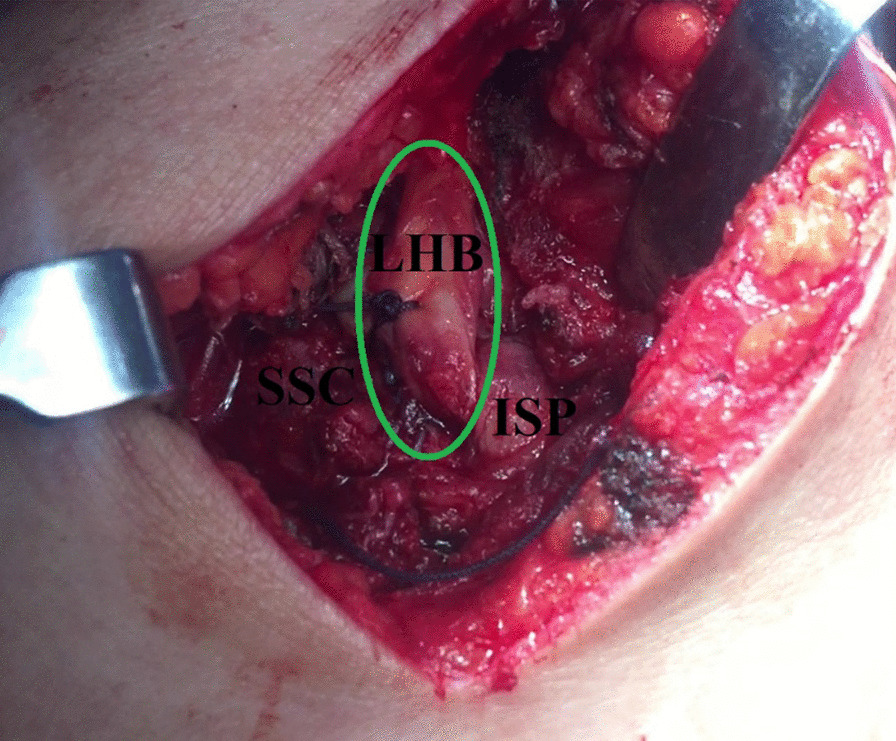
Demonstrates the sutured free segment of the long head of biceps tendon to the repaired rotator cuff in a left shoulder while seating the patient in a beach-chair position. Following sub-acromial/sub-trapezius retrieval of the free segment of the long head of biceps tendon from the sub-acromial space to the scapular wound, this (green oval-marked) segment is further secured (sutured) to the partially-repaired cuff using #2 absorbable sutures. ISP, infraspinatus tendon; LHB, long head of biceps; SSC, subscapularis tendon

**Fig. 11:**
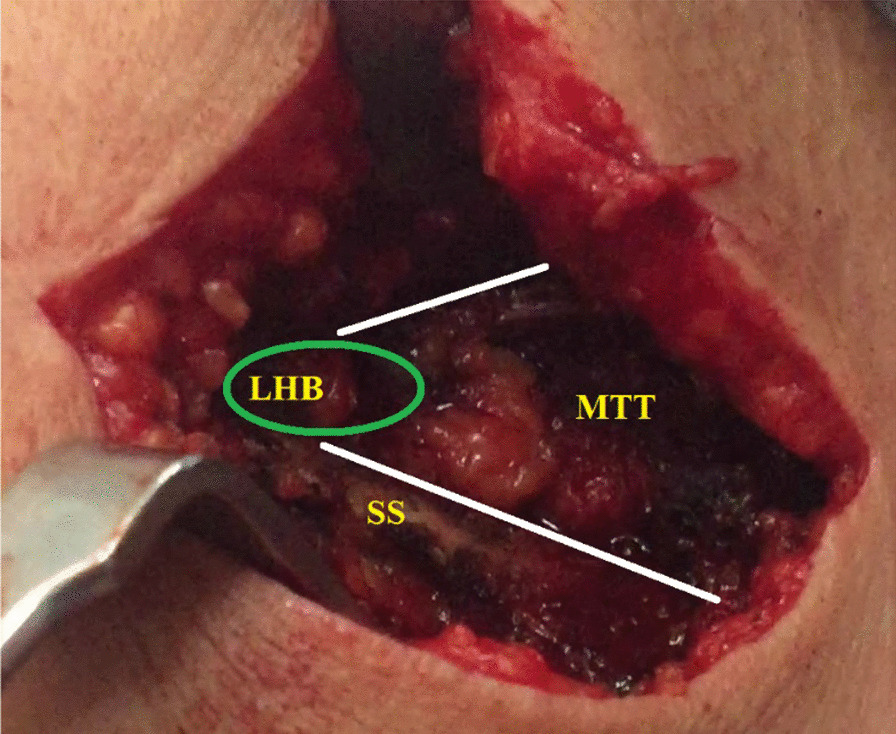
Demonstrates the sutured segment of the long head of biceps tendon to the released middle trapezius tendon in a left shoulder while seating the patient in a beach-chair position. While placing the shoulder in 45°–45° abduction-external rotation, the (green oval-marked) retrieved free segment of the long head of biceps tendon is side-to-side sutured to the released insertion tendon of the medial portion of the middle trapezius muscle (outlined with white lines) using #5 non-absorbable sutures. LHB, long head of biceps; MTT, middle trapezius tendon; SS, scapular spine

(A) The skin overlying medial part of the scapular spine is transversely incised starting from the medial scapular border and extending laterally for 7–8 cm. Subcutaneous tissue is incised in line with skin incision; and swept-off using a dry gauze sponge in order to expose the most medial 8–10 cm of insertion tendon of the middle trapezius segment. The latter insertion is tagged with #2 absorbable sutures and released from the scapular spine using a diathermy probe. Then, the released MTT is bluntly dissected from the underlying SSP to maximize excursion of the released tendon. Figure 8 demonstrates the released medial portion of the MTT.

(B) A long straight artery clamp is then passed through the scapular approach running above the partially repaired RC to appear at the sub-acromial space to establish a sub-acromial/sub-trapezius corridor for retrieval of the free segment of the LHB tendon from the sub-acromial space to the scapular wound. Figure 9 demonstrates sub-acromial/sub-trapezius retrieval of the free segment of the LHB tendon from the sub-acromial space to the scapular wound.

(C) On the humeral side, the retrieved free segment of the LHB tendon is sutured to the partially-repaired cuff using #2 absorbable sutures. Figure 10 demonstrates the sutured free segment of the LHB tendon to the repaired RC.

(D) While placing the shoulder in 45°–45° abduction-external rotation, the retrieved free segment of the LHB tendon is sutured to the released MTT (in side-to-side fashion) using #5 non-absorbable sutures (Ethibond*Excel, Ethicon, Cincinnati, OH, USA). Figure 11 demonstrates the sutured segment of the LHB tendon to the released MTT.

### Dynamic assessment of the biceps-based 3-layer RC reconstruct

Via placing the operated shoulder in different GH positions of elevation/rotation, integrity and smooth sub-acromial motion of the tendon reconstruct are dynamically evaluated. Pearls and pitfalls of the currently reported technique of biceps-based 3-layer RC reconstruction are summarized in Table 1.

**Table 1:** Pearls and pitfalls of the currently reported technique of biceps-based 3-layer rotator cuff reconstruction

*Pearls*	
Arthroscopic GH examination: for exclusion of labral detachment/lesions of the LHB tendon; and associated intra-articular pathology (e.g., arthritic changes) Sub-pectoral LHB tenotomy: performed distally as much as possible to maximize the length of the harvested LHB tendon Dimensions of the created trough: at least 1.5 cm in length, 1 cm in width and 0.5 cm in depth to adequately accommodate the transposed LHB tendon Double-row LHB tenodesis: to ensure distribution of the stresses over 2 different fixation points; and maximize the contact surface area and pressure at the LHB tendon/bone interface Side-to-side suturing of the repaired cuff to the LHB tendon: for re-enforcement of partial repair of the cuff; and near-total coverage of the humeral head Release of MTT: extended laterally as much as possible to maximize length of the harvested tendon (not less than 8–10 cm) Excursion of released MTT: improved via extensive dissection (up to the medial scapular border) of the released MTT from the underlying SSP Side-to-side suturing of MTT to LHB tendon: performed while placing the shoulder in 45°–45° abduction-external rotation	
*Pitalls*	
Prior to LHB tenotomy, the tendon should be well-cleaned off from the surrounding tissues; and well-isolated over an artery clamp to avoid iatrogenic injury to the nearby structures Anatomic repair of SSC is essential for GH re-stabilization via the transverse force couple mechanism Avoid implantation of the suture anchors into the commonly-encountered humeral cysts; otherwise, the whole construct might be at a higher risk of failure Prior to MTT harvesting, meticulous hemostasis is a must for facilitated harvesting of MTT tendon (and its related periosteum) with adequate length and width	

*GH* gleno-humeral, *LHB* long head of biceps, *MTT* middle trapezius tendon, *SSC* subscapularis

For more detailed illustration, technical principle and steps of the currently reported technique of biceps-based 3-layer tendon RC reconstruction are demonstrated in Additional file 1: Video Legend, Additional file 2: Video S1.

## Postoperative rehabilitation

For 6 weeks, the operated shoulder is placed in a regular shoulder immobilizer; however, active within-the-sling cis-cross shoulder exercises are encouraged. Afterwards, the immobilizer is discontinued allowing the patient to resume his light daily-living activities and start pendulum and assisted-active elevation/rotation exercises for 2 weeks. By the 8th postoperative week, a rehabilitation protocol under co-supervision of the surgeon and a physiotherapist is commenced. That protocol includes 2–4 weeks of passive ROM (stretching) exercises followed with 2–4 weeks of active (strengthening) exercises followed with 2–4 weeks of neuromuscular coordination exercises. Return to heavy-duty/overhead/sports activities is allowed by the 5–6th postoperative month.

## Discussion

In a recent publication, Kandeel reported favorable postoperative outcomes of a 3-layer tendon reconstruct (i.e., augmentation of in-situ SCR-reinforced partial RC repair with hamstring-sheet-lengthened MTT transfer) for management of irreparable postero-superior RC tears [13].

For technical simplification, the current note describes a modification of that tendon reconstruct wherein harvesting of the hamstring tendons is avoided; instead, the LHB tendon is used as a corner-stone (i.e., common) structure for in-situ SCR, MTT transfer, and partial RC repair.

### Indications and contra-indications

The currently reported note is described for primary and revision management of deficient postero-superior RC as a result of tear, re-tear or isolated supra-scapular nerve paralysis in middle-aged populations of high functional demands.

It is needless to mention that norm preconditions of tendon transfer (e.g., non-arthritic mobile GH joint) are essential perquisites for the current note. Indications and contraindications of the currently reported technique of biceps-based 3-layer RC reconstruction are summarized in Table 2 [13].

**Table 2:** Indications and contraindications of the currently reported technique of biceps-based 3-layer rotator cuff reconstruction

*Indications*	
Irreparable RC tear RC re-tear Isolated supra-scapular nerve injury	
*Contraindications*	
*Absolute contraindications	
SLAP lesion destabilizing superior labrum-biceps anchor complex Tearing/rupture of LHB Advanced arthritis of GH joint Trapezius muscle paralysis Irreparable SSC Irreparable ISP Non-functioning deltoid muscle (e.g., dehiscence, axillary nerve injury) Active infection	
*Relative contraindications	
History of GH infection Un-motivated patient for 6–9 months postoperative rehabilitation Elderly patients (i.e., > 65 years old) Shoulder stiffness	

*GH* gleno-humeral, *ISP* infraspinatus, *LHB* long head of biceps, *MTT* middle trapezius tendon, *RC* rotator cuff, *SSC* subscapularis, *SLAP* superior labrum from anterior to posterior [12, 13]

### Technical considerations

According to a cadaveric study of Denard et al., the mean diameter of the LHB tendon at the articular margin of the humeral head; and at the biceps musculo-tendentious junction was 6.6 mm; and 5.3 mm respectively. As well, the mean length of that tendon from its labral origin to the articular margin of the humeral head; and to the lower border of PM was 2.5 cm; and 11.5 cm respectively [14].

In the current technical note, the utilized LHB tendon stump can be divided into 3 segments; the proximal segment (2.5 cm) utilized for in-situ SCR, and anchorage of the partially-repaired postero-superior RC; the middle segment (1.5–2 cm) utilized for double-row LHB tenodesis into the created trough; and the distal segment (7–7.5 cm) utilized as a lengthening graft for the transferred MTT.

As regards the 1st layer of the reconstruct, dimensions of the created trough should be at least 1.5 cm in length, 1 cm in width and 0.5 cm in depth in order to adequately accommodate the transposed LHB tendon; hence, inherent stability of LHB tenodesis is promoted [2–4, 13].

In the current description, LHB tendon was fixed into the trough via double-row tenodesis to ensure distribution of the stresses over 2 different fixation points. The proximal anchor is to withstand SCR-related stresses; whereas, the MTT-related stresses are resisted with the distal anchor. In addition, that double-row tenodesis is to maximize the contact surface area and pressure at the LHB tendon/bone interface; thus improving the local biomechanics for tenodesis healing [2, 4].

With respect to the 2nd layer of the reconstruct, partial RC repair is performed taking advantage of uncut sutures of the anchors used for LHB tenodesis; this in turn is to allow the repaired cuff to act as a roof for the created trough; therefore, the inherent stability of LHB tenodesis is further promoted [2, 13].

In terms of the 3rd layer of the reconstruct, distal segment of LHB tendons exhibits geometrical characteristics which might largely differ from those of the hamstring sheet. Technical differences of the currently reported technique of biceps-based 3-layer RC reconstruction from that of recently published 3-layer tendon reconstruction of Kandeel, 2023 are summarized in Table 3 [12–14].

**Table 3:** Technical differences of the currently reported technique of biceps-based 3-layer rotator cuff reconstruction from that of recently published 3-layer tendon reconstruction of Kandeel, 2023

Technical difference	Current technique	Technique of Kandeel, 2023
Biceps tenodesis for in-situ SCR	Double-row tenodesis	Single-row tenodesis
Anatomic landmarks for MTT harvesting	Medial 3/4 of scapular spine	Medial half of scapular spine
Approach for MTT harvesting	8 cm skin incision over and parallel to medial 3/4 of the scapular spine	5 cm skin incision over and parallel to medial half of the scapular spine
Released insertion of MTT	Medial 3/4 of its scapular-spine insertion (via sub-periosteal dissection)	Medial half of its scapular-spine insertion (via sub-periosteal dissection)
Extensile harvesting of MTT	Feasible (in lateral direction) *Medially: limited by spinal accessory nerve	Feasible (in lateral direction) *Medially: limited by spinal accessory nerve
Split of fleshy MTT	Oblique split (along its fibers), (for 8–10 cm)	Oblique split (along its fibers), (for 5 cm)
Release of fleshy MTT from underlying SSP	Blunt dissection (finger sweeping)	Blunt dissection (finger sweeping)
Interposition tendon graft	Retrieved LHB (7X0.5 cm) (Shorter and thinner graft)	Fashioned hamstring sheet (12X1.5 cm)
Corridor for interposition graft & transferred MTT	Sub-trapezius/sub-acromial	Sub-trapezius/sub-acromial
Fixation method of transferred MTT to RC footprint	Double-row tenodesis of the LHB into a trough at midportion of the greater tuberosity Direct suturing of the retrieved segment of LHB to repaired RC	Trans-osseous sutures of the hamstring sheet to RC (SSP ± ISP) footprint Direct suturing of the sheet to repaired RC
Reproduction of anatomic attachment of transferred MTT to SSP footprint	Feasible (less reproducible)	Feasible (via double-row/suture-bridge re-attachment of flattened periosteal end of hamstring sheet to SSP footprint)
Re-attachment of released MTT	Sutured (in side-to-side fashion) to the retrieved segment of LHB	Sutured (in Pulvertaft/side-to-side fashion) to the hamstring sheet
Scapular/GH position during reconstruction	Retracted scapula & 45°-abduction/45°-external rotation of GH joint	Retracted scapula and 45°-abduction/45°-external rotation of GH joint
Room for gliding motion of the tendon reconstruct	SSP fossa and SSP outlet	SSP fossa & SSP outlet
Mechanical block of the tendon reconstruct	–	–
AC joint injury	–	–
Force vector of the transferred MTT	Horizontally-oriented (medially-directed)	Horizontally-oriented (medially-directed)

*AC* acromio-clavicular, *GH* gleno-humeral, *ISP* infraspinatus, *LHB* long head of biceps, *MTT* middle trapezius tendon, *RC* rotator cuff, *SCR* superior capsular reconstruction, *SSP* supraspinatus [12–14]

### Biomechanical considerations

**Fig. 12:**
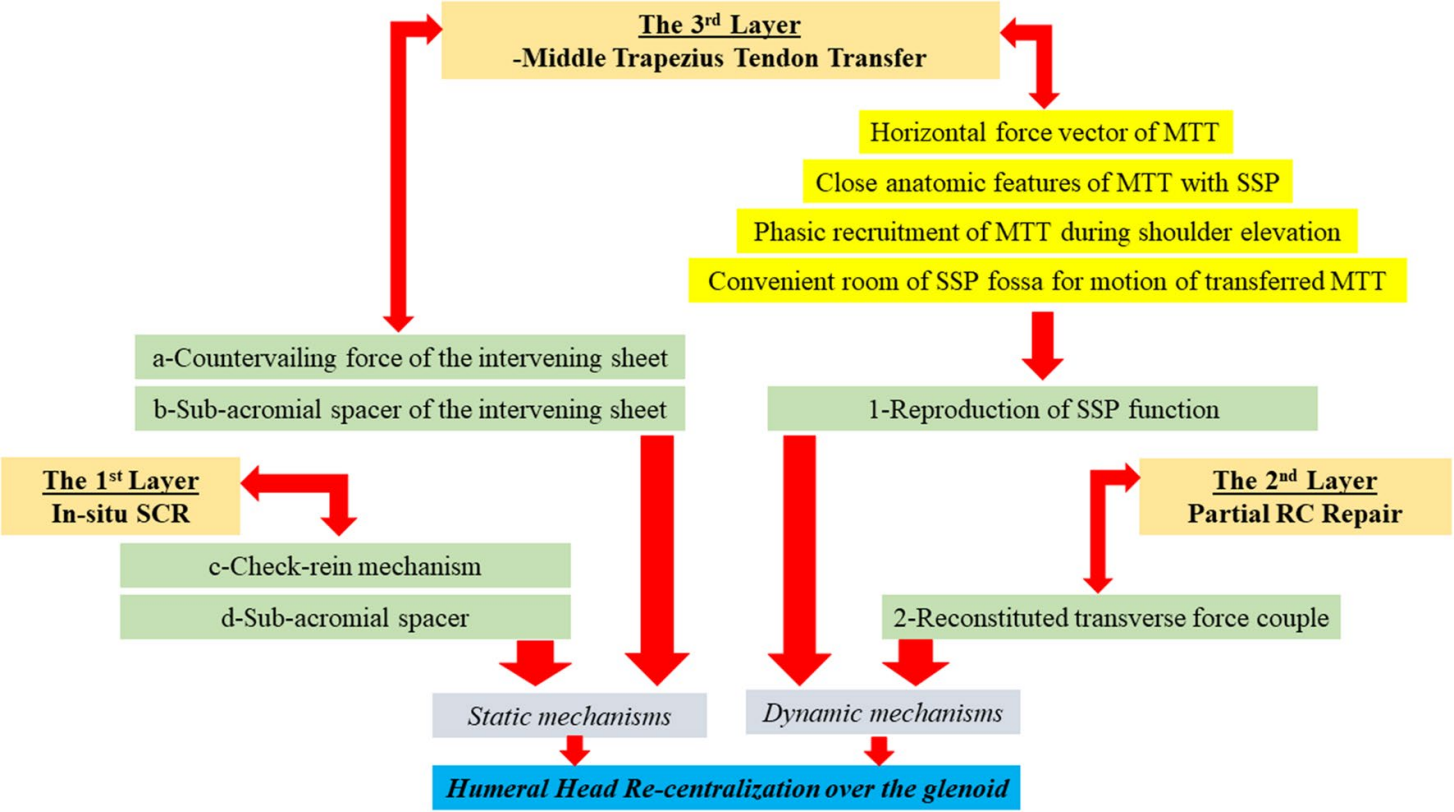
Demonstrates a summary of the biomechanical mechanisms of the currently reported technique of biceps-based 3-layer rotator cuff reconstruction for re-centralization of the humeral head over the glenoid; MTT, middle trapezius tendon; RC, rotator cuff; SCR, superior capsular reconstruction; SSP, supraspinatus [10, 12, 13]

In spite of this technical modification, the current biceps-based 3-layer RC reconstruction is to keep the different static and dynamic mechanisms previously reported (in the original description of MTT transfer) to almost normalize GH kinematics. Figure 12 demonstrates a summary of the biomechanical mechanisms of the currently reported technique of biceps-based 3-layer RC reconstruction for re-centralization of the humeral head over the glenoid [10, 12, 13].

### Biological considerations

From a biological perspective, the highly vascularized transferred middle trapezius in conjunction with the preserved labral attachment of LHB tendon are to ensure optimized biological environment for healing of the tendon reconstruct [1–4, 13, 15].

### Potential advantages

The current technical modification might be rationalized with the advantages of use of LHB tendon (e.g., local availability, ready attachment to the superior glenoid/labrum, and preserved proprioception and vasculature). In addition, the hamstring graft-related drawbacks such as prolonged operative time (for graft harvesting, preparation and fashioning), troublesome setup/positioning, and donor-site morbidity are avoided [1–4, 12, 13].

Besides, the current note might offer the advantages of technical simplicity, and reproducibility; and familiarity of shoulder surgeons with most of its surgical steps (e.g., biceps tenodesis; sub-pectoral exploration for the LHB tendon).

Moreover, the current note can be further modified to be performed via arthroscopic-assisted approach (e.g., in-situ SCR, sub-acromial space retrieval of the LHB tendon from the sub-pectoral region, and shuttling of the LHB tendon to the scapular wound).

### Technical limitations

The current note might herald its own limitations; e.g., technical irreducibility in patients with SLAP lesions destabilizing the superior labrum-biceps anchor complex; or extensive tearing, or rupture of the LHB tendon [2, 4, 13].

Another limitation might be that mucinous degeneration of the proximal LHB tendon is relatively common in the patient populations addressed with the current note; possibly, this degeneration might result in higher postoperative pain scores, and greater risk of structural/biological failure of the tendon reconstruct [16].

It is worth mentioning that the distal segment of the LHB tendon offers a relatively shorter (i.e., 7–7.5 cm) intervening graft compared with that (12 cm) of the fashioned hamstring sheet used in the original description of MTT transfer. To overcome that technical limitation; LHB tendon should be distally-tenotomized as much as possible to maximize length of the intervening graft. Furthermore, length of the released MTT should not be less than 8–10 cm; and that released tendon should be extensively dissected from the underlying SSP up to the medial scapular border in order to improve excursion of the transferred tendon. These technical pearls are essential steps for minimizing the displacement of the trapezius-related neuro-vasculatures; and reducing the tension stresses across the LHB-MTT interface; hence, risk of failure of the tendon reconstruct is lowered [12–14].

As a result of smaller width (5.3–6.6 mm) of the LHB tendon compared with that (15 mm) of the fashioned hamstring sheet; the current modification is less likely to completely cover the humeral head compared with its hamstring sheet counterpart. This limitation might be partly overcome with side-to-side suturing of the postero-superior RC/SSC to the the in-situ reconstructed superior capsule [12–14].

A further limitation might be the use of anchors for dual tasks (i.e., biceps tenodesis and partial RC repair) which might result in a relatively troublesome suture management; and stress over-loading of the anchors; the latter might predispose the tendon reconstruct for failure.

Moreover, a point of debate might be questionable reduction in power of elbow flexion/forearm supination following sub-pectoral biceps tenotomy (for LHB tendon harvesting); and consequently, some could recommend re-attaching the distal biceps to a nearby structure (e.g., PM).

However, according to different reports, this point should not raise a major concern. In a case–control study investigating power of elbow flexion/forearm supination following biceps tenotomy, Shank et al. demonstrated comparable outcomes between the operated and the sound limbs. Also in a recent meta-analysis, Shang et al. concluded insignificant differences between biceps tenotomy and tenodesis in terms of elbow flexion/forearm supination strength indices [17, 18].

Advantages and disadvantages of the currently reported technique of biceps-based 3-layer RC reconstruction are summarized in Table 4.

**Table 4:** Advantages and disadvantages of the currently reported technique of biceps-based 3-layer rotator cuff reconstruction

*Advantages*	
Use of LHB as a common graft (local availability, ready attachment to the superior glenoid/labrum, and preserved proprioception and vasculature) Avoidance of the hamstring graft-related drawbacks such as prolonged operative time (for graft harvesting, preparation and fashioning), troublesome setup/positioning, and donor-site morbidity Technical simplicity, familiarity, and reproducibility Feasibility of arthroscopic-assisted modality of the technique Re-normalized GH kinematics (re-centralized humeral head over the glenoid) via different static and dynamic mechanisms Preservation of sound scapular kinematics Avoidance of mechanical block of the tendon reconstruct Versatility of the indications Accelerated postoperative rehabilitation Relatively-easy revision	
*Limitations*	
Technical irreproducibility in trapezius muscle paralysis, detached superior labrum-biceps anchor complex, or extensive tearing/rupture of LHB Relatively-short intervening/interposition LHB graft for the transferred MTT Incomplete coverage of the humeral head (relatively-thin LHB graft) Use of anchors for dual tasks (stress overloading of the anchors) Multiple re-attachment interfaces Possible seroma formation Relatively higher risk of post-operative infection No biomechanical validation No related electrophysiological verification No long-term cohort clinical studies Unclear biomechanical consequences on nearby cervical spine	

*GH* gleno-humeral, *LHB* long head of biceps tendon, *MTT* middle *trapezius* tendon [12, 13]

## Conclusion

To conclude, for management of irreparable postero-superior RC tears, the currently-reported technical note of biceps-based 3-layer tendon reconstruction might offer the advantages of reproducibility, safety, simplicity and quickness simplicity. As well, it avoids the hamstring graft-related complications. However, it should be validated via further biomechanical and clinical studies.

## Supplementary Information

**Additional file 1.** Video Legend.

**Additional file 2.** Video S1.

## Data Availability

Materials of the current work are available as a Additional file 2 (video form).

## Abbreviations

AC: Acromioclavicular
GH: Gleno-humeral
LHB: Long head of biceps
MTT: Middle trapezius tendon
PM: Pectoralis major
RC: Rotator cuff
ROM: Range of motion
SCR: Superior capsular reconstruction
SSC: Subscapularis
SSP: Supraspinatus
