# Supplementary material for: Biceps-based 3-layer reconstruction of the irreparable rotator cuff: a technical note on biceps tendon as a common local graft for in-situ superior capsular reconstruction, partial cuff repair, and middle trapezius tendon transfer
Source: J Orthop Surg Res. 2023 Jul 15;18:499. doi: 10.1186/s13018-023-03978-0 (PMC10349402; doi:10.1186/s13018-023-03978-0)
Supplement: Supplementary file 1 — Additional file 1. Video Legend. [file 13018_2023_3978_MOESM1_ESM.docx]

**Supplemental Video Legend**

-This video is to demonstrate a technical note on use of the long head of biceps (LHB) tendon as a common local graft for in-situ superior capsular reconstruction (SCR), partial rotator cuff (RC) repair, and middle trapezius tendon (MTT) transfer for management of irreparable RC tears.

- Kandeel has recently introduced the transfer of medial (lower) portion of the MTT for (dynamic) reproduction of the supraspinatus (SSP) function; which in turn is to result in effective re-centralization of the humeral head over the glenoid.

- This biomechanical rationale of MTT transfer was investigated in a recently-published cohort study which revealed that a 3-layer tendon reconstruct (in which partially-repaired RC was augmented on its articular side with in-situ SCR; and on its bursal side, with hamstring-tendon-lengthened MTT transfer) has shown more superior postoperative functional outcomes compared with a 2-layer tendon reconstruct of in-situ SCR-reinforced partial RC repair for management of irreparable postero-superior RC tears.

- However, a major technical default of MTT transfer is the need for an intervening sheet of hamstring tendons auto-graft to lengthen the transferred MTT tendon to the native RC footprint. Graft-related complications such as prolonged operative time, troublesome setup/patient positioning, higher risk of infection and donor site morbidity might hinder popularization of that currently-evolving transfer. **^(13)^**

- For technical simplification and effective reproducibility of that 3-layer tendon reconstruct, the current note describes a biceps-based 3-layer RC reconstruction in which LHB tendon is simultaneously used as a local graft for in-situ SCR; an interposition sheet (as an alternate to the hamstring tendons autograft) to lengthen the transferred MTT; and an anchorage structure for partial RC repair.

- The prime indications of the current note include primary and revision management of irreparable postero-superior RC tears in relatively-young active population with high functional demands.

- On the other hand, the current note is absolutely contra-indicated in patients with SLAP lesions destabilizing the superior labrum-biceps anchor complex; or extensive tearing, or rupture of the LHB tendon. Other contra-indications include trapezius muscle paralysis and active GH infection.

- The current note is performed while seating the patient in beach-chair position. Following pen-marking of the related anatomic landmarks, passive range of motion (ROM) of the shoulder is evaluated in order to exclude associated shoulder stiffness.

- Afterwards, diagnostic arthroscopy is performed to confirm the diagnosis of RC irreparability, ascertain intact/reparable SSC, assess integrity of the labral attachment/intra-articular portion of LHB, and preclude associated intra-articular GH pathology (e.g., arthritic changes).

- Via McKenzie approach, the sub-acromial space is decompressed; and integrity of the proximal LHB tendon is assessed.

- Then, via a sub-pectoral approach; the LHB tendon is identified, lifted up over a curved artery clamp; tagged with #2 absorbable sutures, cleaned off from the surrounding soft tissues along down the tendon as distally as possible to maximize the length of the harvested tendon, and tenotomized distal to the tagging sutures. Using a long straight artery clamp, the proximal stump of the LHB tendon is then retrieved from the sub-pectoral region to the sub-acromial space.

- Thereafter, an osteotome and a mallet are used to create a trough over the mid-portion of the greater tuberosity. Into this trough, two suture anchors are sequentially inserted into its proximal and distal portions in order to complete a double-row biceps tenodesis for reconstitution of the superior GH capsule taking advantage of the glenoid-attached proximal segment of the LHB tendon while leaving the tendon portion distal to the tenodesis site as a free segment for future use in biceps-based MTT transfer.

- Next, postero-superior RC is partially-repaired taking advantage of uncut free suture limbs of the anchors used for biceps tenodesis, this partial repair is further reinforced with 3-4 side-to-side simple stitches annexing the LHB tendon and the partially-repaired RC together.

-Through a 7-8cm transverse skin incision over the medial part of the scapular spine, the most medial 8-10cm of insertion tendon of the middle trapezius segment is identified, tagged with absorbable sutures and released from the scapular spine using a diathermy probe. Then, the released MTT is bluntly dissected from the underlying SSP to maximize excursion of the released tendon.

- Afterwards, a long straight artery clamp is passed through the scapular approach running above the partially repaired RC to appear at the sub-acromial space to establish a sub-acromial/sub-trapezius corridor for retrieval of the free segment of the LHB tendon from the sub-acromial space to the scapular wound.

- On the humeral side, the retrieved free segment of the LHB tendon is sutured to the partially-repaired RC using #2 absorbable sutures.

- While placing the shoulder in 45^O^-45^O^ abduction-external rotation, the retrieved free segment of the LHB tendon is sutured to the released MTT (in side-to-side fashion) using #5 non-absorbable sutures

- Via placing the operated shoulder in different GH positions of elevation/rotation, integrity and smooth sub-acromial motion of the tendon reconstruct are dynamically evaluated.

-As regards postoperative rehabilitation, the operated shoulder is placed in a regular shoulder immobilizer for 6 weeks followed with a 3-month tri-phasic program of stretching, strengthening and neuro-muscular coordination exercises. Return to heavy-duty/overhead/sports activities is allowed by the 5-6^th^ postoperative month.

- In spite of this technical modification, the current note is to keep the different dynamic and static mechanisms previously reported (in the original description of MTT transfer) to almost normalize GH kinematics; including dynamic reproduction of SSP function, and static restraint of superior migration of the humeral head via the countervailing force, check-rein mechanism, and sub-acromial spacer effect of the interposition graft and the reconstructed superior capsule.

- From a biological perspective, the highly vascularized transferred middle trapezius in conjunction with the preserved labral attachment of LHB tendon are to ensure optimized biological environment for healing of the tendon reconstruct.

- Technically, while avoiding the hamstring graft-related complications; use of LHB tendon as a local graft offers the advantages of local availability, ready attachment to the superior glenoid/labrum, and preserved proprioception/vasculature.

- Otherwise; the current note is not without limitations. One of these limitations might be the relatively-short and thin interposition graft of LHB tendon used for lengthening the transferred MTT. Another limitation might be that mucinous degeneration of the proximal LHB tendon is relatively common in the patient populations addressed with the current note; consequently, this degeneration might result in higher postoperative pain scores, and greater risk of structural/biological failure of the tendon reconstruct.

- For management of irreparable postero-superior RC tears, the currently-reported technical note of biceps-based 3-layer tendon reconstruction might offer the advantages of reproducibility, safety, quickness, and simplicity. As well, it avoids the hamstring use-related complications. However, it should be validated via further biomechanical and clinical studies.
